# Supplementary material for: UnidecNMR: automatic peak detection for NMR spectra in 1-4 dimensions
Source: Nat Commun. 2025 Jan 7;16:449. doi: 10.1038/s41467-024-54899-3 (PMC11707349; doi:10.1038/s41467-024-54899-3)
Supplement: Supplementary file 1 — Supplementary Information [file 41467_2024_54899_MOESM1_ESM.pdf]

Supplementary information for:

UnidecNMR: Automatic peak detection for NMR spectra in 1-4 dimensions

Charles Buchanan[1,2], Gogulan Karunanithy[1], Olga Tkachenko[1], Michael Barber[1], Michael Marty[1,3], Timothy Nott[4], Christina Redfield[4], Andrew J. Baldwin[1,2*]

[1]Physical and Theoretical Chemistry, University of Oxford, South Parks Road, Oxford, UK

[2]Kavli Institute for Nanoscience Discovery, Sherrington Rd, Oxford, UK

[3]Department of Chemistry and Biochemistry, University of Arizona, Tuscon Arizona, USA

[4]Department of Biochemistry, University of Oxford, South Parks Road, Oxford University, UK

Correspondence to be addressed: andrew.baldwin@chem.ox.ac.uk

Contents

Supplementary Figure 1: The results of UnidecNMR are relatively tolerant to the choice of peak shape.

Supplementary Figure 2: Raw HNCA of aB-crystallin

Supplementary Figure 3: The UnidecNMR GUI

Supplementary Figure 4: Optimisation of UnidecNMR algorithm in 1D

Supplementary Figure 5: Investigation of the missing UnidecNMR 2D peak

Supplementary Figure 6: Comparison of performance in 2D on DDx4

Supplementary Figure 7: Analysis of UnidecNMR errors in DDX4 data

Supplementary Figure 8: Accuracy of identified peak positions for 'correctly' picked peaks in simulated 2D data

Supplementary Figure 9: Accuracy of UnidecNMR intensities of 'correctly' picked peaks in 2D

Supplementary Table 1: Results of tested peak picking algorithms against 2D and 3D experimental data from proteins.

Supplementary Table 2: Detailed NMRPipe processing parameters for the analysed protein spectra

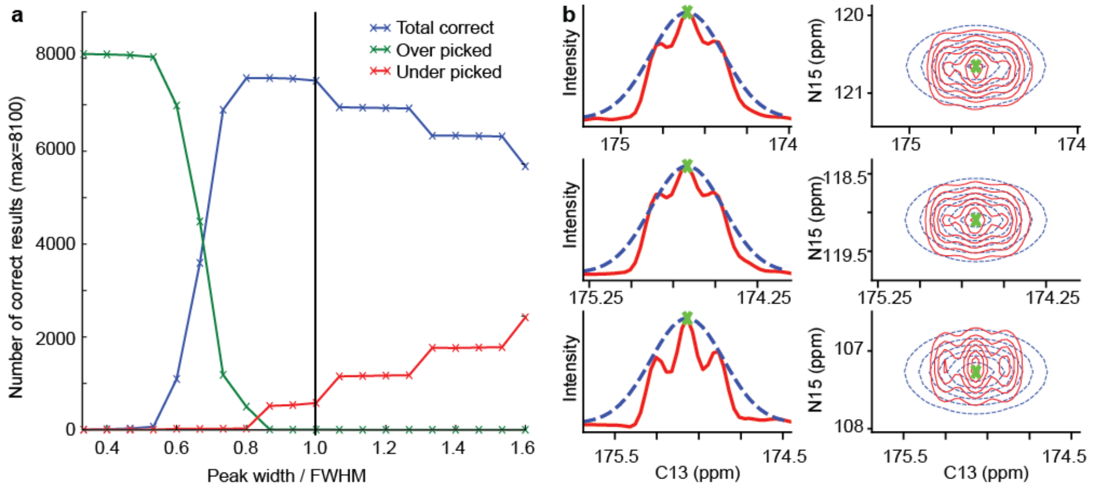

Supplementary Figure 1: The results of UnidecNMR are relatively tolerant to the choice of peak shape.

a The accuracy of 2D peak picking against the simulated dataset (Fig. 2). Maximum accuracy was achieved If the peak width used for calculation was between 0.8 and 1 of the 'actual' peak width. When the peak width is set to be lower than this, the algorithm will over-pick the spectrum. When the peak width is set to be wider than this, the algorithm tends to under-pick, with the success rates falling off gradually. The discrete form of the plot is due to the 'squash' window being specified in the program as an integer of points. In cases where peak widths are substantially wider than this, UnidecNMR can be run twice with different settings for the peak shape.

b In the HNCO spectrum from HSP16.5, the decoupling was mis-set and the peak shape in the ^{13}C dimension resembles a triplet. While undesirable, this spectrum can be accurately analysed using UnidecNMR by setting the peak shape to be broad enough to encompass the triplet. The other software tested in this paper were unable to reliably assign this spectrum (Table 1).

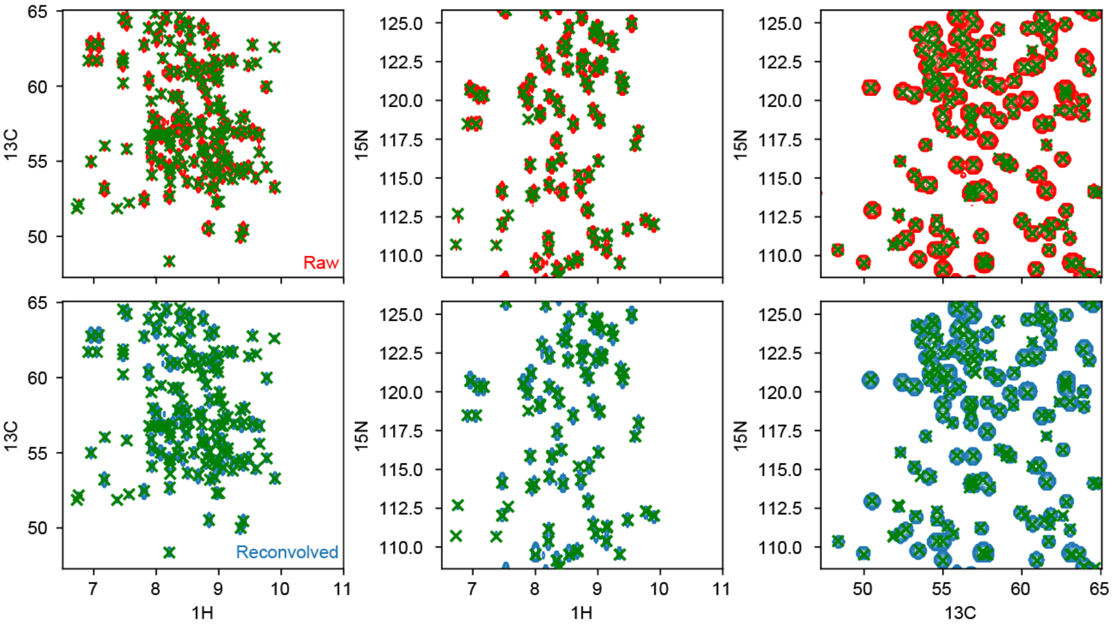

Supplementary Figure S2: Raw HNCA of aB-crystallin (Red) shown in projection with the reconvolved result from UnidecNMR (blue) and the picked peaks (green). For analysing 3D data, 'fac' in UnidecNMR was set to 1.6.

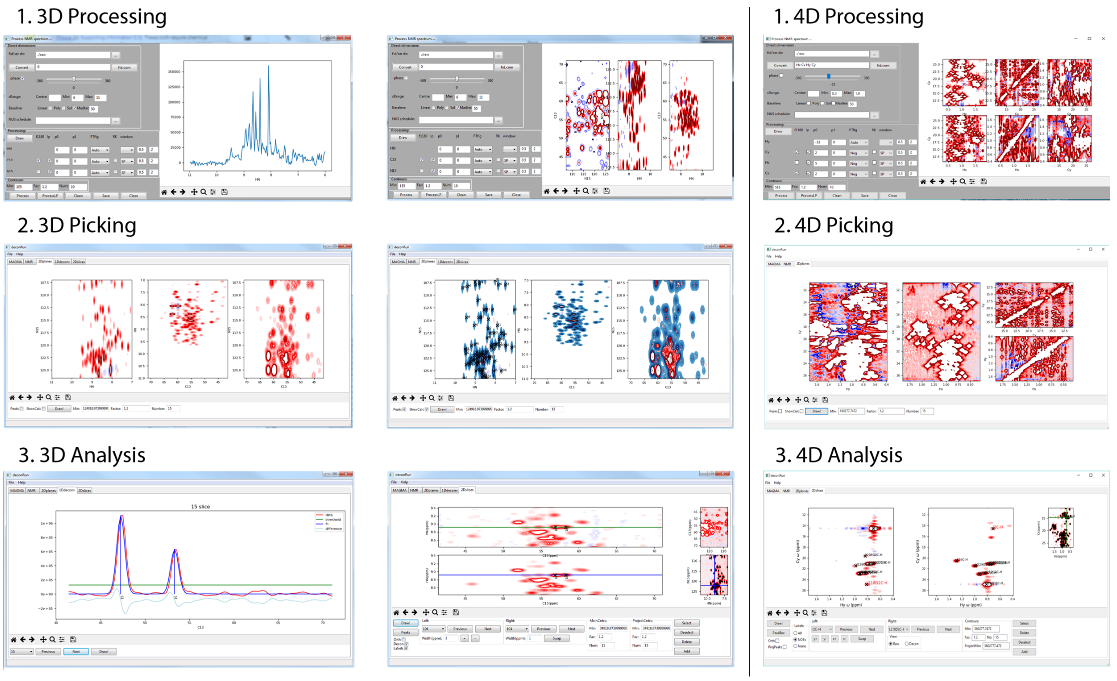

Supplementary Figure 3: The UnidecNMR GUI. Screen-shots of the GUI that we release with Unidec. **a)** Raw 3D and 4D NMR spectra can be interactively processed. The GUI allows a user to specify options which it uses to generate and execute nmrPipe scripts. Projections of the spectrum are calculated and displayed to allow iterative editing. Selecting the resolution enhancement option will generate a script using the SMILE algorithm. Specifying a file in the non-uniform sampling box will generate and execute SMILE based scripts for spectral reconstruction. **b)** The projected planes, together with the back-calculated spectra from UnidecNMR can be compared. C) After executing the spectral deconvolution, the raw data and the back-calculated results can be directly compared in 1 and 2 dimensions. The program aims to show users where the current selected plane is within the nD hypercube by showing the location on projections. Tutorial videos are available on YouTube.

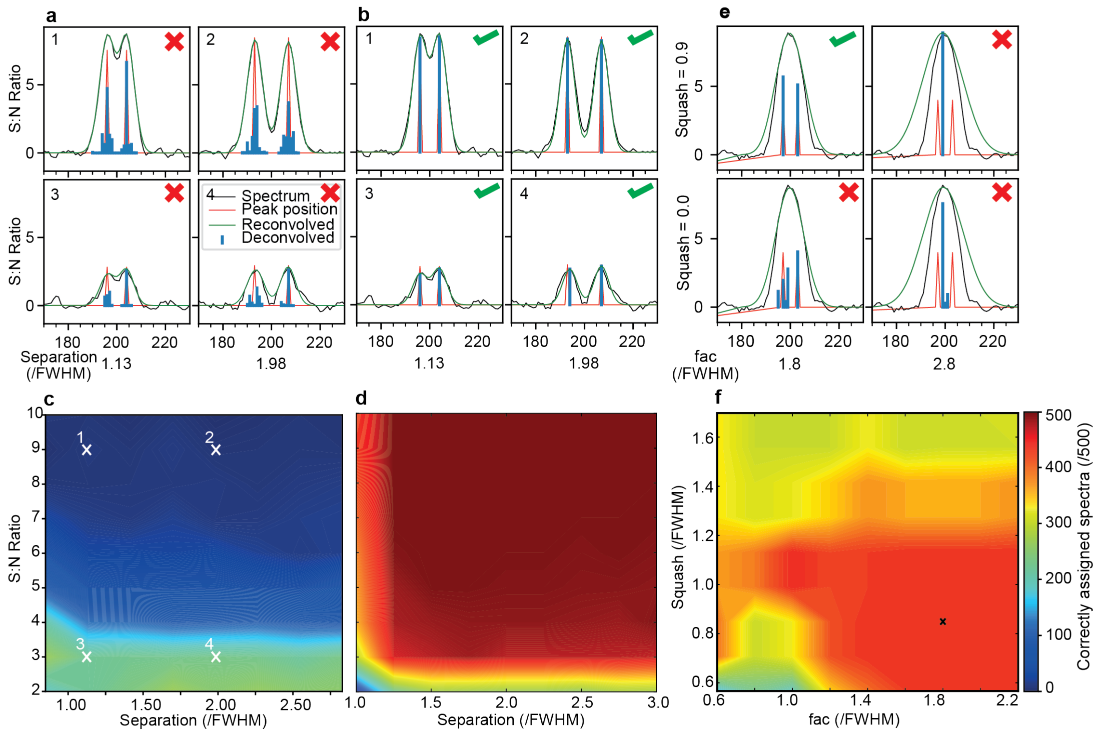

Supplementary Figure 4: Optimisation of UnidecNMR in 1D.

To assess and develop the naïve Unidec algorithm into UnidecNMR, we simulated 1D NMR spectra arranged over a 9x9 grid of peak separation, for a range of signal to noise values (see extended methods). For each point, 500 spectra were individually created making a 'complete' test dataset of 40.5k simulated spectra. **(A)** Prior to optimisation, the naïve Unidec algorithm 'over-picked' resonances as shown on 4 spectra. **(C)** Applying the naïve Unidec algorithm to the full dataset demonstrates that it is not effective. The peak detection was defined as 'correct' if exactly two peaks were picked within 0.5 FWHM of the locations simulated. At best the naïve algorithm was successful for 1/5 spectra even in the high S/N, large separation (the 'easy') limit. **(B)** after refinement, the performance of the UnidecNMR is outstanding. The same specimen spectra as shown in (A) are now correct, and (D) the success of the algorithm is close to 100% provided that peaks are higher than S/N of 3, and separated by 1.25 of the FWHM. Below these limits, the ability of the algorithm to function depends on the precise interaction between noise and the underlying spectrum. **(C)** As described in the text, to handle the overpicking, there are two stages to the optimisation of the algorithm. The first is that the calculation is first run using an artificially inflated filter shape, whose peak width is increased by a factor 'fac'. The second is that a simple clustering algorithm is applied, where all intensity within a window, defined in terms of the FWHM of the filter shape, is combined and placed at the most intense location (squash window). With an optimised combination of 'fac' and 'squash' (1.8, 0.9 respectively), peak detection works very well (top left). With the 'squash' clustering turned off (bottom row), the algorithm overpicks resonances. With the 'fac' width multiplier set to be too high (2.8), the algorithm under-picks resonances (top right). **(F)** Taking the 500 spectra from a heavily overlapped region (0.8 separation, 8 S/N) we assessed the performance of the algorithm for a range of the variables 'squash' and 'fac'. A broad plateau corresponding to fac between 1.4 and 2.2, and squash 0.6 to 1.0 resulted in perfect performance. These values are physically intuitive. For squash, it is reasonable to cluster intensity below one FWHM as our ability to resolve resonances better than this should be impossible. For fac, as shown in (and (E), too small and we overpick, and too larger and we underpick.

For the final distribution of the algorithm, optimal performance is obtained for squash set to 0.75 FHWM in all cases, and the value of fac is left to be a user controlled parameter. Optimal performance is seen with fac=1.4 for 1D and 2D, and 1.6 for 3D.

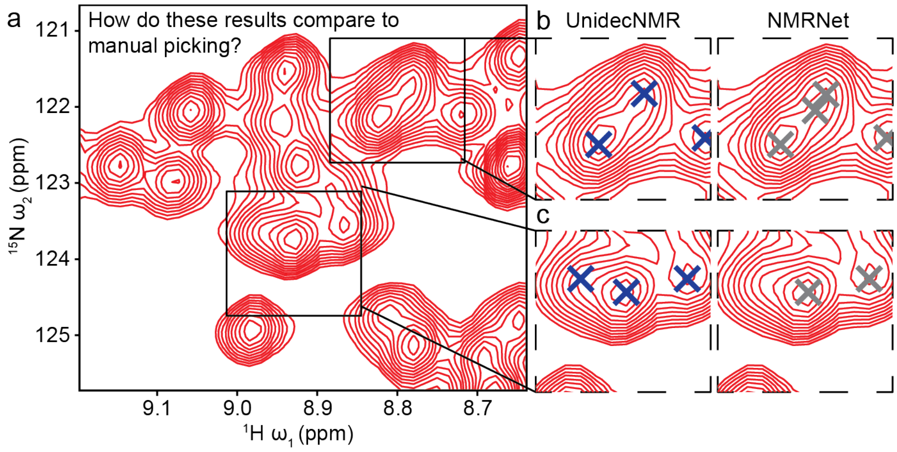

Supplementary Figure 5: Investigation of the missing UnidecNMR 2D peak.

A region of an ^{15}N HSQC spectrum of αB-crystallin showing significant overlap (a). Two challenging regions are isolated (b,c) with the results of two algorithms indicated. (b) We believe that a skilled user would identify 3 resonances based on inspection of this data alone. However, we know when we analyse the corresponding HNCO that in case b, there are 4 resonances. For the purposes of scoring, because NMRNet correctly identified 4 peaks in this region, we counted this example as a case where UnidecNMR missed a peak and NMRNet was completely correct. (c) In this case, a skilled user would pick 3 resonances. These were correctly identified by UnidecNMR, but one was missed by NMRNet. We conclude that the performance of UnidecNMR is consistent with what a skilled user would identify when presented only with this spectrum, but in the interests of fairness, we nevertheless scored the missed peak in b as an error. This is the '1 missed peak' in the HSQCs in Fig. 4a.

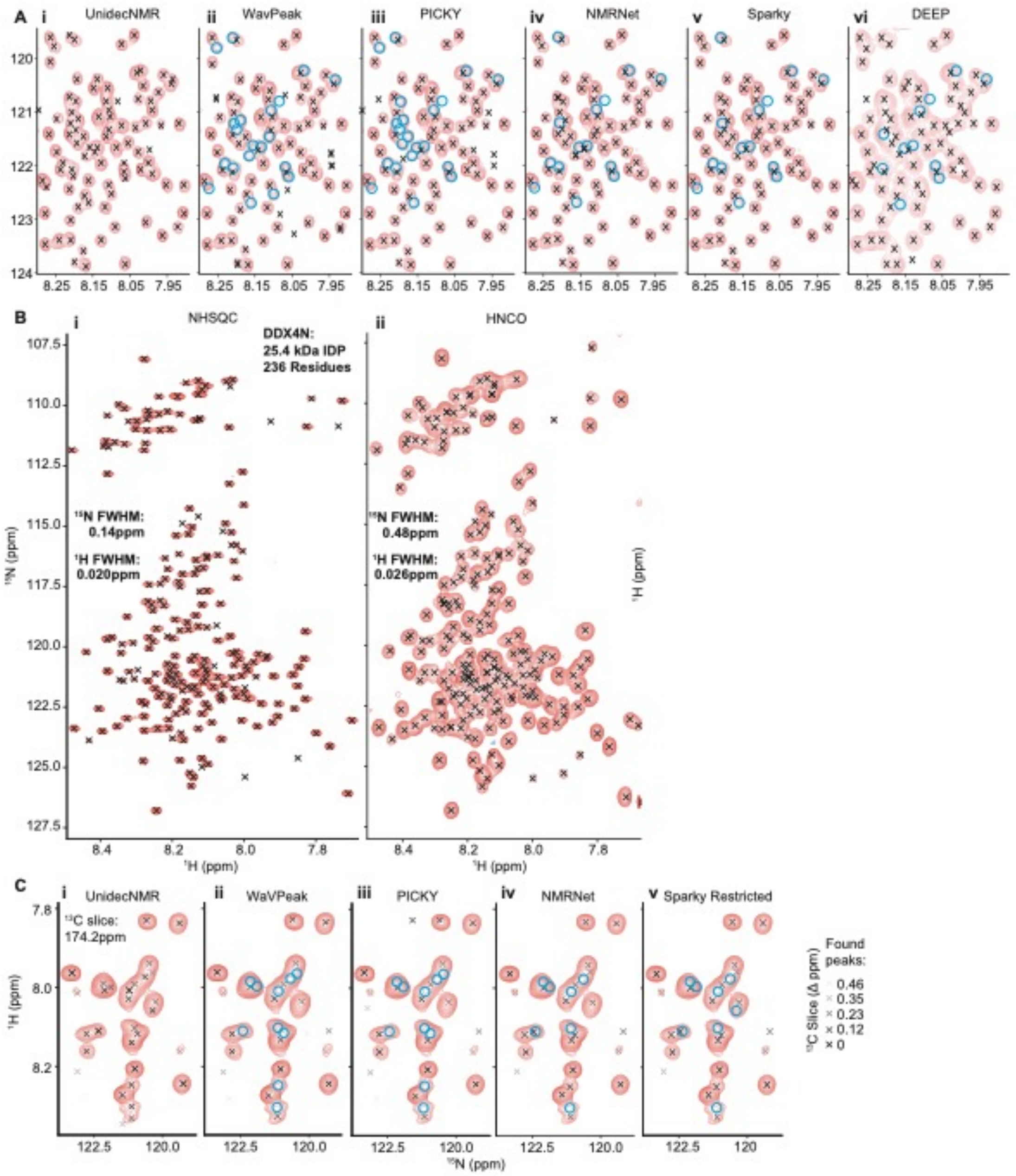

Supplementary Figure 6: Comparison of performance in 2D on DDx4

A more detailed comparison of the different peak pickers tested on data acquired at 750 MHz from the 236 intrinsically disordered protein DDX4N1. **A:** A comparison of the different peak picking methods on the high resolution 2D ^{15}N HSQC. Identified peaks are shown with black crosses, while false negatives are shown with blue circles. UnidecNMR is able to identify far more subtlety within the spectrum. A Lorentz-to-gauss window function was used for all data processing except for the DEEP algorithm. In this work, it is recommended that a 2-pi kaiser window function in their instructions (NMRPipe command 'SP -off 0.5 -end 0.896 -pow 3.684') is applied (https://github.com/lidawei1975/deep). Performance of DEEP when run on the data as processed for the others was improved by running on a spectrum processed this way versus the processing used for the other spectra. Even when analysing this heavily overlapped spectrum, performance of UnidecNMR is similar to that of an experienced user selecting peaks by hand whereas performance for the others was noticeably less good. For UnidecNMR, 'fac' was set to 1.4, the standard value for 2D deconvolution. **B:** A comparison of a high resolution ^{15}N HSQC, and the H-N projection of the HNCO, with superimposed peak lists. The comparison shows clearly difference in resolution between spectra,

which helps illustrate why analysing the 3D using the ‘boring’ mode is so effective. The HNCO peaks shown here were easily mapped across from the higher resolution NHSQC using the UnidecNMR GUI.

C: A slice from the HNCO highlights the ability of UnidecNMR to unpick overlapped spectra when compared to the other algorithms. Various shades of grey are used to indicate different position^{s} in the 3*rd* (*13*C) dimension relative to the focused $^{15}N/^{1}H$ slice indicated. UnidecNMR is again able to perform as well as a manual user here by employing Bayesian logic and mapping the higher resolution NHSQC spectrum across to this lower resolution spectrum. In 3D, as described in the detailed methods, the variable fac for the UnidecNMR algorithm is set to 1.6.

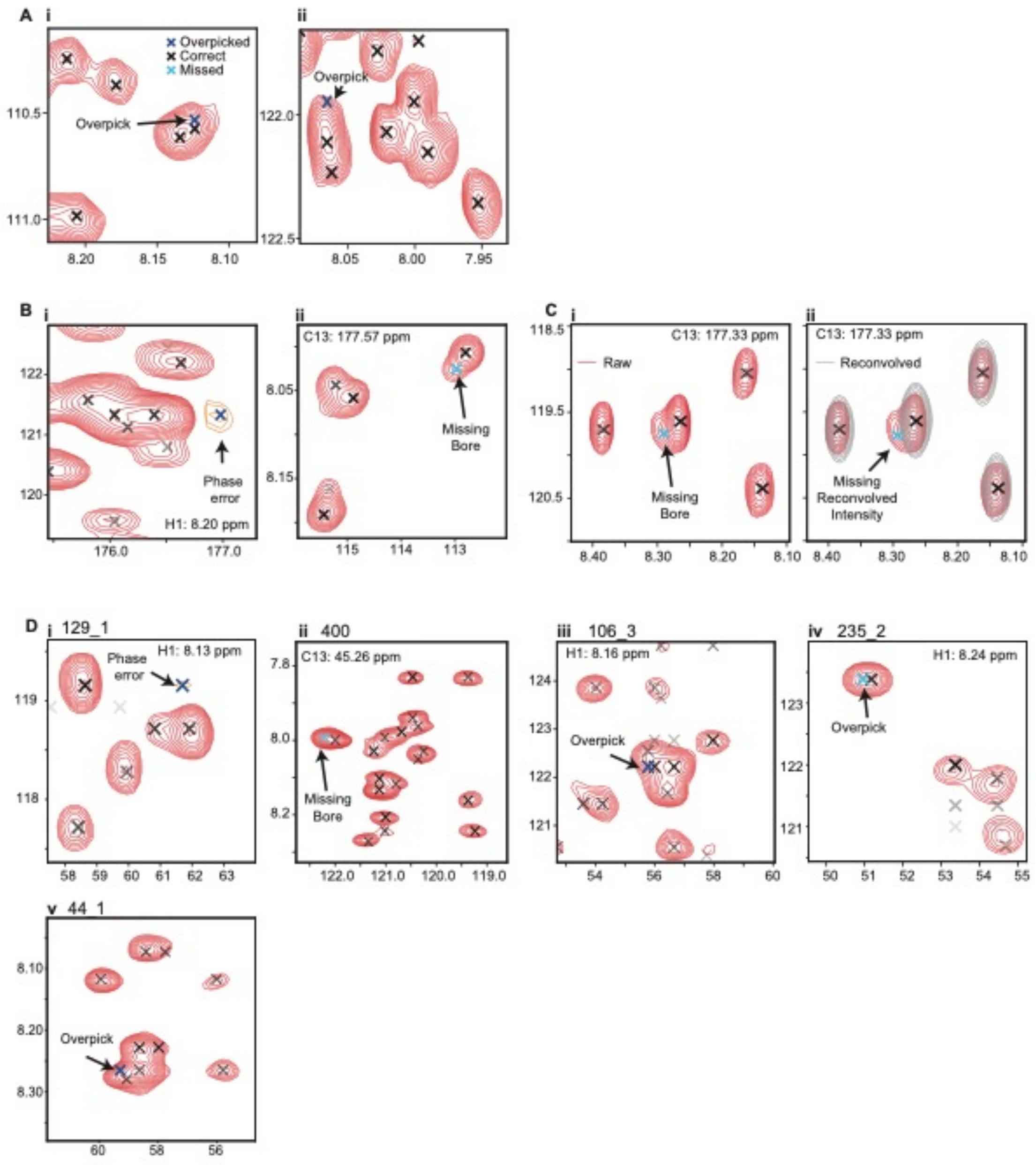

Supplementary Figure 7: Analysis of UnidecNMR errors in DDX4 data A detailed exploration of the errors encountered when analysing DDX4N1 data. UnidecNMR parameter 'fac' was set to 1.4 for 2D and 1.6 for 3D. Overall, there were 10 errors when compared to a manual analysis, comprising three different classes, 'overpick' x 5, 'missing bores' x 3 and negative intensity artefacts x2.

The negative intensity artefacts are easily spotted and removed manually, occurring in regions of the spectra where we do not expect resonances from proteins.

The 'missing bores' result from us using the 'boring' mode when analysing data. Here, a 2D peak list is first acquired from a 15N HSQC spectrum, as described in the text. This list is then used to restrain a 3D analysis. There were 3 cases where resonances were observed in the HN plane of HNCA/HNCO spectra, but did not show up in the HSQC. Performance in general is enhanced by using the 'boring' mode, but these are cases where it fails. Using the GUI, it is straightforward to add these resonances in. These resonances are correctly identified by running the program in straight 3D where boring is not used, and the correct number of correlations in 3D are identified in this region.

The 5 overpicks are cases where in a heavily overlapped region, the program supplies a false positive typically on the edge of a resonance. We conservatively define these as errors on the basis that analysis of these spectra in isolation, a skilled user would not confidently pick these.

A: 2D 15N HSQC: two 'overpicks'. No evidence for these resonances were found in the 3D spectra.

B: HNCO: **i)** A negative artefact occurring close to an intense peak, easily identified and removed using the included GUI. **ii)** A 'missing bore' not picked in the HSQC. Two resonances are clearly found in 3D. The error is rapidly revealed through a manual inspection, and both resonances are identified when running the peak picker in full 3D.

C: HNCO: **i)** a second 'missing bore'. **ii)** When the reconvolved spectrum is shown together with the raw one, the difference is very clear and easily corrected manually.

D: HNCA: a negative artefact, a third missing bore and 3 overpicks.

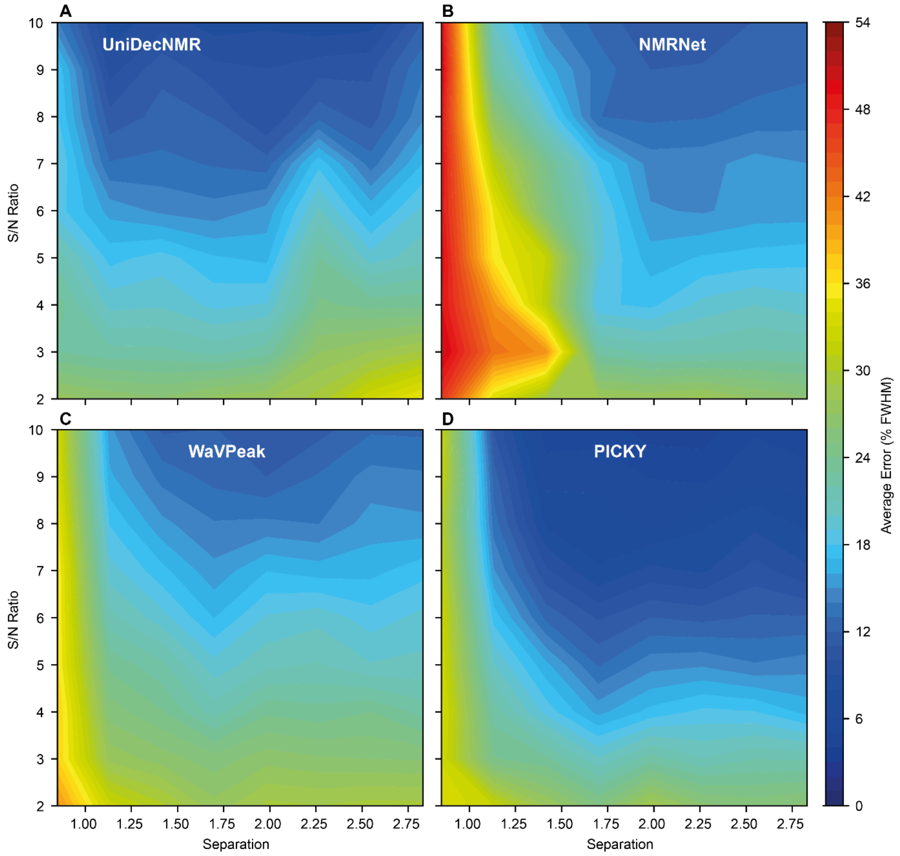

Supplementary Figure 8: Accuracy of identified peak positions for 'correctly' picked peaks in simulated 2D data

For peaks defined identified as 'correct' in the 2D simulated spectra, we quantified the average deviation from the detected position to the known location. The average value for the ca. 500 spectra per grid point is shown, expressed in terms of FWHM units. For UnidecNMR **(A)**, this value is between 10% (SN > 6) and ca. 30% (SN<6). Similar performance was found for both Picky and WaVPeak. The accuracy of NMRNet was similar although it was notably less accurate for closely separated resonances than the other algorithms.

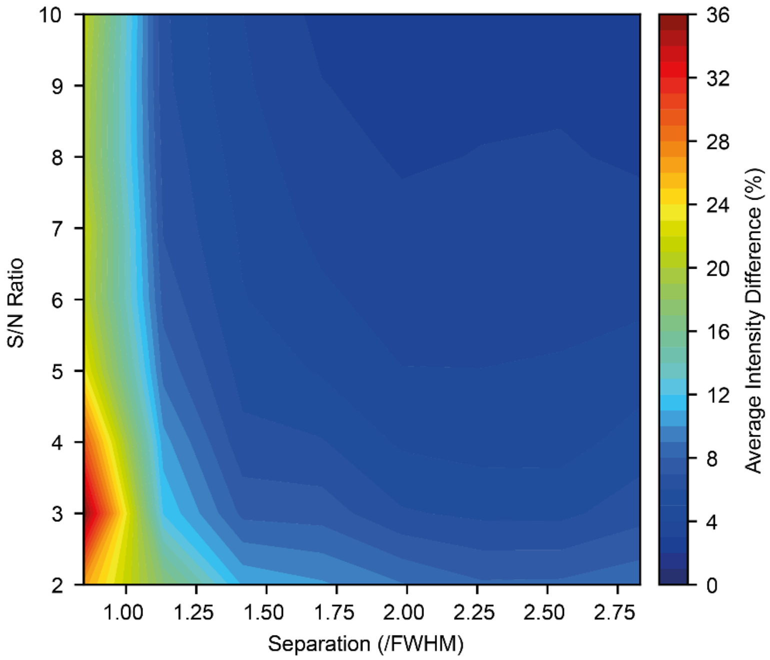

Supplementary Figure 9: Accuracy of UnidecNMR intensities of 'correctly' picked peaks in 2D

Intensities are not given for resonances for the other programs tested. For resonances returned by UnidecNMR we were able to assess the accuracy of returned intensities by comparing the relative intensities returned by the two simulated resonances. As these were both simulated to be identical, any deviation in their relative intensity indicates that the returned intensities are unreliable. Provided that the intensities are separated by more than 1.25 of a FWHM, the intensities returned for correct peaks is better than 5%. In cases where resonances are heavily overlapped, the uncertainty in the intensity increases to ca. 20% for S/N > 5.

Protein	Type	Manual Backbone	UnidecNMR	NMRNet	PICKY	WaVPeak	Sparky
HSP27	NHSQC	75	**75/11/0/7**	72/7/0/8	69/10/3/5	70/9/13/7	72/9/3/12
(2x10kDa)	HNCO	79	**79/7/0/4**	78/8/5/1	75/3/7/0	75/6/14/3	
	HNCA	147	**147/1/1/3**	139/2/6/0	130/0/15/1	129/0/58/2	
Ubiquitin	NHSQC	71	**71/18/0/0**	71/18/0/0	70/18/5/0	71/18/1/0	71/18/3/0
(8kDa)	HNCO	71	**71/3/0/0**	71/0/0/0	71/0/0/0	71/7/7/0	
	HNCA	139	**139/0/0/1**	139/3/3/5	138/0/2/4	138/16/22/2	
αBcrystallin	NHSQC	79	**78/10/1/0**	78/10/3/1	67/8/16/0	73/8/16/0	78/10/0/0
(2x10kDa)	HNCO	80	**80/0/0/0**	79/0/5/3	73/0/10/5	73/0/16/4	
	HNCA	145	**145/0/1/6**	133/0/18/7	115/0/39/6	115/0/59/7	

Supplementary Table 1: Results of tested peak picking algorithms against 2D and 3D experimental data from proteins.

To enable a detailed comparison between the algorithms in 2 and 3D, we first compiled a reference set of peak picks for three proteins, as summarised in Fig 4. The difficulty of the three range from 'easy' (ubiquitin) where all resonances are very clear and well resolved, 'medium' (20 kDa HSP27) where the majority of resonances are well resolved with some exhibiting exchange broadening and 'hard' (20 kDa αB-crystallin) where exchange broadening leads to a range of intensities and peak shapes. Spectra tested were 2D 15N/1H HSQC, and 3D HNCO and HNCA.

Identifying 'correct' and 'incorrect' peak picks can be subjective. For the purposes of this scoring exercise, we focused on an objective, and practical definition for 'correct peaks'. We manually peak picked the spectra, and compared the results to known backbone assignments, validated with multiple other 3D assignment spectra.

In the HSP27 and aB-crystallin spectra, the 'correct' number of resonances in HSQC is smaller than that in the HNCO due to overlap, and we are taking care here to define 'correct' peak picking as that which a skilled user would do if given only this spectrum in isolation. In the HNCA, we expect two peaks per observed HN minus 1. Low signal to noise and overlap prevented us from reaching this value. When combined with additional data during assignment, this overlap can be clearly resolved, but this is an impossible expectation from a peak picker given only this spectrum.

As these spectra are based on the detection of HN pairs, we expect to see resonances from side chains as well as from the backbone. Side chain resonances can be identified from their chemical shifts, and are typically present in 3D spectra at aliased chemical shifts, and have complex peak shapes, isotope shifts and coupling patterns. We did not penalise any of the algorithms on their performance on these resonances, which experimentally can be identified and removed from consideration by acquiring a single unaliased HSQC spectrum. All algorithms identified peaks in the vicinity of side chain resonances, but we were unable to define a reliable 'gold standard' success rate for these.

Further, resonances that were manually identified in only one of the three spectra, and wasn't a side chain resonance, were classified as 'ambiguous'. These are resonances that would be picked by a skilled user if investigating these spectra in isolation but could not be reliably assigned. Algorithms were not penalised for picking or missing a resonance in this category, and these were not scored as 'false positives' for any algorithm. These were all resonances with low signal intensity.

A 'false positive' we define as a resonance picked by any of the algorithms that would not be reasonably picked by a skilled user.

The results of each test are tabulated as A/B/C/D, as
A: correctly assigned backbone resonances
B: correctly assigned side chain resonances (associated with Gln, Asn, often aliased in 3D spectra, easily distinguished from the backbone, these have been excluded from correct and incorrect counts)
C: False positives: resonances that would not be picked by a skilled user.
D: ambiguous: weak resonances that are not clearly from side chains, that appear in only 1 of the three spectra that a skilled user would identify as a resonance, but we were unable to unambiguously assign.

The false negatives for any spectrum can be calculated from the manual backbone count, minus the 'correct' backbone count.

UnidecNMR was run unrestricted on the 2D NHSQC spectrum. The side chain resonances were removed, and the resulting peak list used for picking the 3D HNCO in 'boring' mode, as described in the text. The peak list was updated to analyse the 3D HNCA spectra where 'bores' were found to contain more than one resonance in the HNCO. This procedure was automated, and closely follows how a user would perform an analysis on 3D NMR data. It is not possible to perform this logical restriction with the other peak pickers tested.

UnidecNMR produces the best results for each spectrum. NMRNet, PICKY and WAVPeak perform similarly well for some spectra (Ubiquitin, HSP27 HNCO), but poorly on the more challenging spectra (HSP27 HNCA, αB-crystallin). Sparky, which can only perform 2D, greatly overpicks the spectra in all but ubiquitin. The included GUI makes it easy to identify any errors produced by UnidecNMR.

We scored UnidecNMR as having made 1 false negative in the HSQC of αB-crystallin (Supplementary Figure 5). In brief, we make an exception here as would not expect this resonance to be picked by a skilled user owing to its overlap with another. However, NMRNet picked two resonances in this region for this spectrum. While we are unsure why NMRNet made this decision, we cannot fault it, and we elect to count this as a success for NMRNet, and an error for UnidecNMR.

Sample	Spectrum	NUS	Apodisation Function	SOL	Zero Filling
αB-crystallin	NHSQC	No	Sine-Bell pow 1 off 0.5 end 0.98	Yes	Yes
	HNCO	No	Sine-Bell pow 2 off 0.5 end 0.99	No	Yes
	HNCA	No	Sine-Bell pow 2 off 0.5 end 0.99	Yes	Yes
HSP27core	NHSQC	No	Sine-Bell pow 2 off 0.5 end 0.99	No	Double
	HNCO	No	Sine-Bell pow 2 off 0.5 end 0.98	Yes	Yes (F3, direct dimension), Double (F2,F1)
	HNCA	No	Sine-Bell pow 2 off 0.5 end 0.98	Yes	Yes (F3, direct dimension), Double (F2,F1)
Ubiquitin	NHSQC	No	Sine-Bell pow 1 in F2 (1H), pow 3 in F1 (13C), off 0.5 end 1.0 in both	No	Yes
	HNCO	No	Sine-Bell pow 2 off 0.5 end 0.99	No	Yes
	HNCA	No	Sine-Bell pow 2 off 0.5 end 0.99	No	Yes
DDX4N1	BEST-TROSY-NHSQC	No	Lorentz-to-gauss GM NMR pipe function:<br>F2 (1H): g1=3.0, g2=5.0, g3=0.0<br>F1 (15N): g1=0.0, g2=8.0, g3=0.0	Yes	Double
	BEST-TROSY-HNCO	Yes	GM: F3 (1H): g1=0.0, g2=10.0, g3=0.0 F2 (15N): g1=0.0, g2=20, g3=0.0 F1 (13C): g1 = 0.0, g2 = 50, g3 = 0.0	Yes	Double
	BEST-TROSY-HNCA	Yes	GM: F3 (1H): g1=3.0, g2=13.0, g3=0.0 F2 (15N): g1=0.0, g2=13.0, g3=0.0 F3 (13C): g1 =0.0, g2=80.0, g3=0.0	Yes	Double
ATCaseDimer	3D CH3 NOESY	No	Sine-Bell pow 2, off 0.5 end 0.98	No	Double
EIN	4D CH3 NOESY	No	Sine-Bell pow 2, off 0.5 end 0.98	No	Double

Supplementary Table 2: Detailed NMRPipe processing parameters for the analysed protein spectra

All spectra were also phased in their respective dimensions using the UnidecNMR GUI before optional enhancement via digital solvent suppression, apodisation and zero filling. Different spectra were processed in slightly different ways reflecting variations in individual preferences and/or the default from the macro editor in nmrDraw. The peak-picking results are largely independent of the choice of fitting parameters and choice of apodisation function. In cases where NUS is not used, linear prediction is performed using SMILE to double the length of the indirect dimensions. Specific nmrPipe processing commands for the various options are as follows:

NUS: Non-uniform sampling, if yes, reconstruction with SMILE, script generated by UnidecNMR GUI.
SOL: Digital solvent suppression. (-SOL)
ZF: Zero filling: Yes: doubles the data size twice to enhance the apparent digital resolution (-ZF 2). Double, doubles the size of the indirect dimension (-ZF -auto).
