# Supplementary material for: UnidecNMR: automatic peak detection for NMR spectra in 1-4 dimensions
Source: Nat Commun. 2025 Jan 7;16:449. doi: 10.1038/s41467-024-54899-3 (PMC11707349; doi:10.1038/s41467-024-54899-3)
Supplement: Supplementary file 2 — Reporting Summary [file 41467_2024_54899_MOESM2_ESM.pdf]

Corresponding author(s): Andrew Baldwin

Last updated by author(s): Nov 18, 2024

Reporting Summary

Nature Portfolio wishes to improve the reproducibility of the work that we publish. This form provides structure for consistency and transparency in reporting. For further information on Nature Portfolio policies, see our Editorial Policies and the Editorial Policy Checklist.

Statistics

For all statistical analyses, confirm that the following items are present in the figure legend, table legend, main text, or Methods section.

n/a	Confirmed	
☐	☒	The exact sample size (*n*) for each experimental group/condition, given as a discrete number and unit of measurement
☐	☒	A statement on whether measurements were taken from distinct samples or whether the same sample was measured repeatedly
☒	☐	The statistical test(s) used AND whether they are one- or two-sided<br>*Only common tests should be described solely by name; describe more complex techniques in the Methods section.*
☒	☐	A description of all covariates tested
☐	☒	A description of any assumptions or corrections, such as tests of normality and adjustment for multiple comparisons
☒	☐	A full description of the statistical parameters including central tendency (e.g. means) or other basic estimates (e.g. regression coefficient) AND variation (e.g. standard deviation) or associated estimates of uncertainty (e.g. confidence intervals)
☒	☐	For null hypothesis testing, the test statistic (e.g. *F*, *t*, *r*) with confidence intervals, effect sizes, degrees of freedom and *P* value noted<br>*Give P values as exact values whenever suitable.*
☒	☐	For Bayesian analysis, information on the choice of priors and Markov chain Monte Carlo settings
☒	☐	For hierarchical and complex designs, identification of the appropriate level for tests and full reporting of outcomes
☒	☐	Estimates of effect sizes (e.g. Cohen's *d*, Pearson's *r*), indicating how they were calculated

Our web collection on statistics for biologists contains articles on many of the points above.

Software and code

Policy information about availability of computer code

Data collection	The core algorithm is coded in c++. This will be distributed with the software in a compiled form. We clarified that this is acceptable to the journal. The bulk of the code is a graphical interface and linkers to make use of the main binary, this is all open source and written in python. A google drive link has been provided with the code and an example file, together with a you-tube video showing people how to use it all. The software has been downloaded onto nmrbox, an open source host, allowing users to access our software without the need to install anything locally. The core algorithm is identical to the unidec algorithm, designed by us for analysis of native mass spectrometry data as described in the paper. Some adjustment is required to render it suitable for NMR specifically, but all steps necessary are described in detail in the manuscript. There is sufficient information for another scientist programmer to re-create everything we have done should they wish.
Data analysis	All data analysis is described in the paper. But in brief, we have a benchmark dataset consisting of 2D (15N HSQC) and 3D (HNCA/HNCO) spectra from four proteins, DDX4, alphaB-crystallin, HSP27 and Ubiqutin. These were acquired on a range of different spectromters. The benchmark of raw data is provided as a separate download. We have engineered the software so that the entire analysis can be performed with a single command by a user. This will 1) act as a template so users can adapt what already works to their own data and 2) act as a verification that the software performs as described in the paper.

For manuscripts utilizing custom algorithms or software that are central to the research but not yet described in published literature, software must be made available to editors and reviewers. We strongly encourage code deposition in a community repository (e.g. GitHub). See the Nature Portfolio guidelines for submitting code & software for further information.

Data

Policy information about availability of data

All manuscripts must include a data availability statement. This statement should provide the following information, where applicable:

- Accession codes, unique identifiers, or web links for publicly available datasets
- A description of any restrictions on data availability
- For clinical datasets or third party data, please ensure that the statement adheres to our policy

Benchmarking data is available for download from http://UnidecNMR.chem.ox.ac.uk. The 2D HSQC and 3D HNCA/HNCO spectra from the four proteins in the benchmark (ubiquitin, aB-crystallin, hsp27 and DDx4) are present, with input files that allow their processing and peak picking to be performed precisely as described in this manuscript (in conjunction with the program, see code availability statement). These act as a template to allow users to adapt their own data to our environment and as a validation that the software works as described in this manuscript.

Experimental details for the different systems and spectrometer acquisition settings are found in the methods section. Supplementary Table 2 describes detailed settings for processing the FIDs into spectra. Supplementary Table 1 describes how the different peak picking methods were scored.

Research involving human participants, their data, or biological material

Policy information about studies with human participants or human data. See also policy information about sex, gender (identity/presentation), and sexual orientation and race, ethnicity and racism.

Reporting on sex and gender	n/a
Reporting on race, ethnicity, or other socially relevant groupings	n/a
Population characteristics	n/a
Recruitment	n/a
Ethics oversight	n/a

Note that full information on the approval of the study protocol must also be provided in the manuscript.

Field-specific reporting

Please select the one below that is the best fit for your research. If you are not sure, read the appropriate sections before making your selection.

- [x] Life sciences
- [ ] Behavioural & social sciences
- [ ] Ecological, evolutionary & environmental sciences

For a reference copy of the document with all sections, see nature.com/documents/nr-reporting-summary-flat.pdf

Life sciences study design

All studies must disclose on these points even when the disclosure is negative.

Sample size	n/a
Data exclusions	n/a (this is physical science, we don't exclude data!)
Replication	n/a
Randomization	n/a
Blinding	n/a

Reporting for specific materials, systems and methods

We require information from authors about some types of materials, experimental systems and methods used in many studies. Here, indicate whether each material, system or method listed is relevant to your study. If you are not sure if a list item applies to your research, read the appropriate section before selecting a response.

Materials & experimental systems

n/a	Involved in the study
☒	☐ Antibodies
☒	☐ Eukaryotic cell lines
☒	☐ Palaeontology and archaeology
☒	☐ Animals and other organisms
☒	☐ Clinical data
☒	☐ Dual use research of concern
☒	☐ Plants

Methods

n/a	Involved in the study
☒	☐ ChIP-seq
☒	☐ Flow cytometry
☒	☐ MRI-based neuroimaging

Plants

Seed stocks	n/a
Novel plant genotypes	n/a
Authentication	n/a
